# Migratory intralaryngeal thyroglossal duct cyst

**DOI:** 10.4103/0971-3026.63053

**Published:** 2010-05

**Authors:** Pradeep D Karlatti, Swapnil Nagvekar, TP Lekshmi, Abhey S Kothari

**Affiliations:** Department of Radiology, Amala Institute of Medical Sciences, Thrissur, Kerala, India

**Keywords:** Intralaryngeal, migratory, thyroglossal duct cyst

## Abstract

Intralaryngeal thyroglossal duct cysts are rare; a migrating one, rarer still. Such a case may be a cause for confusion and it is important to understand this entity and its typical findings.

## Introduction

Thyroglossal duct cyst is a common cause of median and paramedian swellings of the neck in children.[1,2] These cysts are also common in adults and have been described in supra- and infrahyoid locations.[3,4] We recently saw an intralaryngeal thyroglossal duct cyst, which is a rare occurrence.[4–6]

## Case Report

A 28-year-old male patient presented with a painful left paramedian neck swelling for two months. Physical examination revealed a cystic, mobile swelling in the left paramedian region. The lesion disappeared on hyperextension of the neck and reappeared on lateral rotation to the left side. Routine blood investigations and thyroid function tests were within normal limits.

USG revealed a cystic lesion in the left paramedian aspect of the neck at the level of the thyroid cartilage. Septations and internal echoes were noted within the lesion. Doppler evaluation showed no vascularity within the lesion. The thyroid gland was normal. On hyperextension, the lesion extended into the intralaryngeal region and this was confirmed on CT scan.

**Figure 1 (A-F):**
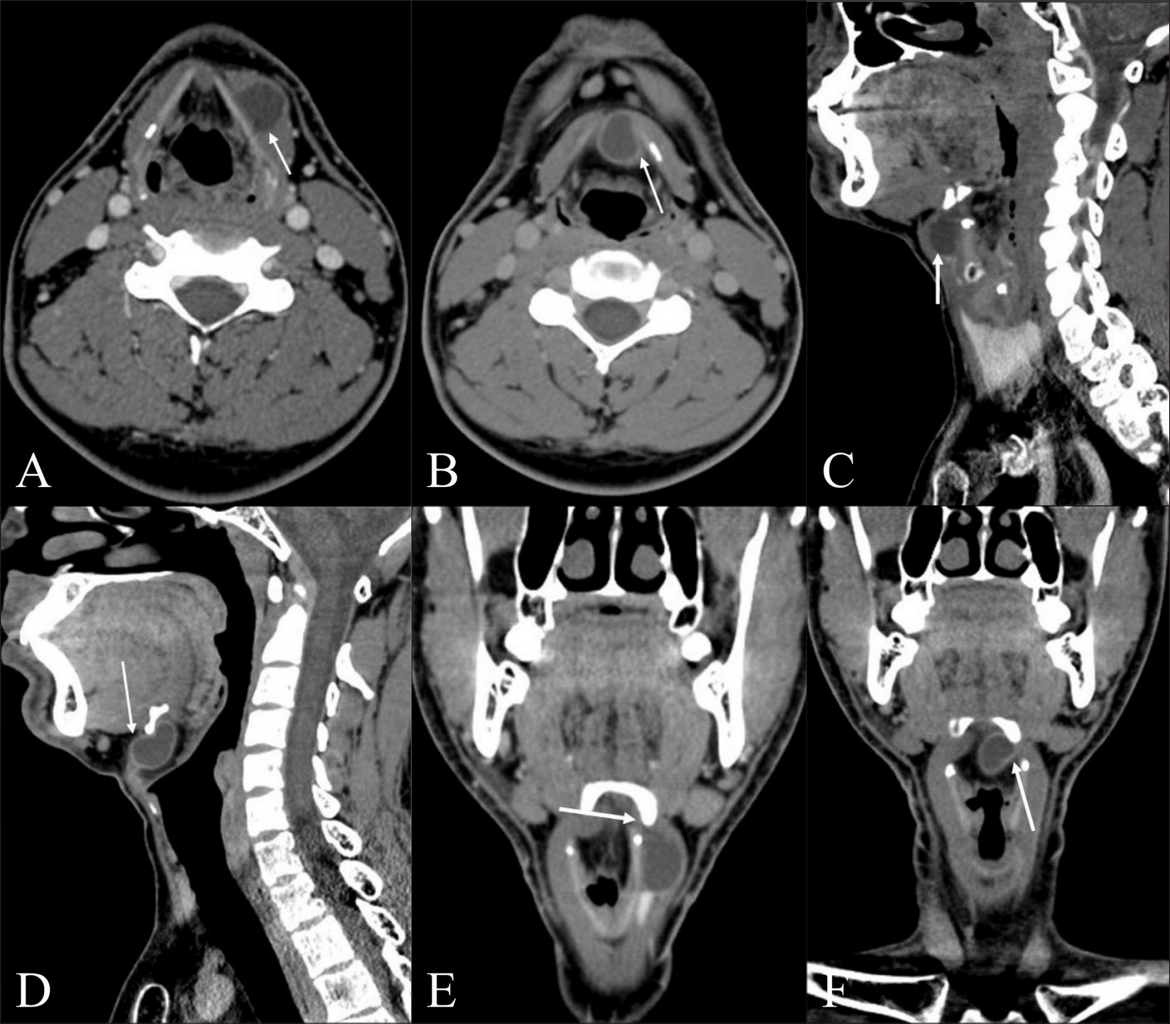
(A) Axial contrast-enhanced CT scans of the neck show a cystic, left paramedian lesion (arrow) located under the the strap muscles closely abutting the thyrohyoid membrane in the neutral position (B) The same lesion assuming an intralaryngeal location after hyperextension of the neck (C) Sagittal reformatted contrast-enhanced CT scans of the neck show a cystic, left paramedian lesion (arrow) located under the the strap muscles closely abutting the thyrohyoid membrane in the neutral position (D) The same lesion assuming an intralaryngeal location after hyperextension of the neck with probable defect in thyrohyoid membrane (E) Coronal reformatted contrast-enhanced CT scans of the neck show a cystic, left paramedian lesion (arrow) located under the the strap muscles closely abutting the thyrohyoid membrane in the neutral position (F) The same lesion assuming an intralaryngeal location after hyperextension of the neck with probable defect in thyrohyoid membrane

CT scan showed a peripherally enhancing cystic lesion under the strap muscles, closely abutting theth thyrohyoid membrane on the left side [Figures 1A, C, and E]. On hyperextension, the cystic lesion was seen migrating into the pre-epiglottic space [Figures 1B, D, and F]. CT scan after the Valsalva maneuver showed coexisting bilateral laryngoceles.

Due to the migratory nature of the lesion, the possibilities of intralaryngeal extension of a thyroglossal duct cyst or extension of a subhyoid bursa through a defect in the thyrohyoid membrane were considered in the differential diagnosis. Surgery revealed a cystic lesion with intralaryngeal extension through a defect in the thyrohyoid membrane. Histopathology confirmed the diagnosis of an inflamed thyroglossal duct cyst.

## Discussion

Though thyroglossal duct cysts are a common differential diagnosis for cystic lesions of the neck in children, they do occur in adults also.[1] These cysts are the remnants of the thyroglossal duct, which gives rise to the thyroid gland.

The thyroid gland arises between the first and second pharyngeal pouch. The initial thyroid precursor is called the thyroid primordium. The thyroid primordium forms an epithelium-lined tubular structure, the thyroglossal duct. It extends from the base of the tongue to the inferior margin of the hyoid bone and at about the seventh week of gestation the thyroid gland completely descends to its final position. By the eighth or tenth week the duct normally undergoes involution or atrophy. The primordial lobe of the thyroid gland may arise from the inferior end of the thyroglossal duct. The failure of any portion of the thyroglossal duct to involute leads to the formation of a thyroglossal duct cyst.[1,2,7]

Thyroglossal duct cysts may be located at the level of the hyoid (15-50%), the suprahyoid (20-25%), or the infrahyoid (25-65%).[1–3] The intralaryngeal location of a thyroglossal duct cyst is rare and only a few cases have been reported.[4–6] In our patient, the cystic lesion was located at the infrahyoid level and there was intralaryngeal migration on movement of the neck, which makes this case interesting.

## Conclusion

Although thyroglossal duct cysts arising from embryonic remnants are located predominantly at the infrahyoid level and are usually extra laryngeal in location, they may also occur in an intralaryngeal location and cause diagnostic difficulties. The present case was doubly confusing because of the migratory nature of the lesion, which was subsequently proven to be an inflamed thyroglossal duct cyst.
